# Analysis of gaps in feline ectoparasiticide purchases from veterinary clinics in the United States

**DOI:** 10.1186/s13071-021-04768-5

**Published:** 2021-05-20

**Authors:** Robert Lavan, Dorothy Normile, Imran Husain, Amita Singh, Kathleen Heaney

**Affiliations:** 1 Center for Observational and Real-World Evidence, Merck & Co., Inc, Kenilworth, NJ USA; 2 Merck Animal Health, 2 Giralda Farms, Madison, NJ USA; 3 Celeritas Solutions LLC, 157 Columbus Avenue, 4th Floor, New York, NY USA; 4 Zickin School of Business, Baruch College CUNY, 55 Lexington Avenue, New York, NY USA; 5 Heaney Veterinary Consulting, LLC, Bradley Beach, NJ USA

**Keywords:** Adherence, Cat, Dosing gap, Ectoparasiticide, Doses plus gap period, Purchase Gap

## Abstract

**Background:**

The study objective was to examine cat owner ectoparasiticide purchases in the United States and estimate the impact of purchase gaps on timely ectoparasite protection administration. These purchase gaps lead to periods of time when cats are unprotected from ectoparasites.

**Methods:**

Ectoparasiticide purchase transactions for individual cats from 671 U.S. veterinary clinics from January 1, 2017 through June 30, 2019 were evaluated to determine time “gaps” between doses of ectoparasiticides purchased in a defined 12-month period. Ectoparasiticides examined were topically applied products that contained fluralaner, fipronil/(S)-methoprene/pyriproxyfen, imidacloprid/pyriproxyfen or selamectin as active ingredients. The duration of protection following administration of one dose was 8–12 weeks for the fluralaner-containing product and one month for the other products.

**Results:**

Ectoparasiticide purchase records were obtained from 114,853 cat owners and analysis found that most owners bought ≤ 6 months of protection during the year, with 61–75% (depending on the product) purchasing just 1–3 months of protection. The size of the average purchase gap was determined for all dose combinations out to 12 months of protection (5–7 doses for fluralaner and 12 doses for the other three products dosed monthly. The largest gaps occurred between the first and second doses and the second and third doses. Average purchase gaps for the four different products between doses 1 and 2 ranged from 11.2 to 13.9 weeks and between doses 2 and 3 ranged from 7.7 to 12.2 weeks. The fraction of purchases separated by gaps and the average length of the gap tended to decrease with increasing number of doses purchased. Owners purchasing the 8 to 12-week duration product containing fluralaner provided ectoparasite protection (“doses plus gap period”) for a larger proportion of each 2-dose period compared with owners purchasing products administered monthly.

**Conclusions:**

When cat owners purchase flea and tick medication, gaps between subsequent purchases reduces the proportion of time ectoparasite protection can be provided. The duration of the gap between doses has an impact on the effectiveness of flea/tick medication because it inserts a period without flea and tick protection between doses of flea and tick medication. The gaps between purchases were shorter and the period of ectoparasite protection was larger for owners purchasing a 12-week product than for owners purchasing a monthly product.

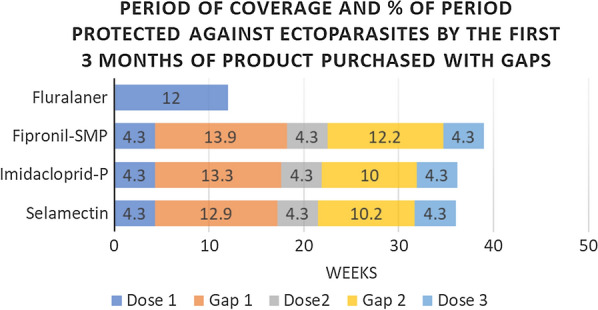

## Background

Fleas, most commonly *Ctenocephalis felis felis*, routinely infest indoor and outdoor residing pet cats, causing irritation, hypersensitivity reactions, and transfer of vector borne pathogens (VBP) [1–8]. Illness inducing VBPs, including *Bartonella* spp., hemoplasmas, and *Rickettsia felis*, with infection rates of up to 80% have been reported in fleas collected from cats [9–11].

Ticks also commonly infest cats causing irritation and transmission of VBP [6, 8, 12]. In a large survey of ticks collected from cats in the USA, various tick species were collected every month of the year, even during winter months in temperate regions. *Ixodes scapularis*, *Amblyomma americanum* and *Dermacentor variabilis* were commonly found, but other species including *A. maculatum, Haemaphysalis longicornis, Otobius megnini,* and less common *Dermacentor spp*. and *Ixodes* spp were collected [13, 14]. Ticks collected from these cats were found infected with a variety of VBP including *Borrelia burgdorferi, Anaplasma phagocytophilum, Ehrlichia chaffeensis* and spotted fever group *Rickettsia* spp. [13]. Some of these tick-borne pathogens are known to infect cats and are associated with clinical illness [10, 11, 15–20]. Surveys of VBP in cats report high to very high prevalence with *Rickettsia* spp. and *Bartonella* spp. being the most predominant [21–24]. These are zoonotic pathogens and carry associated risks for pet owners and veterinarians [16, 25–28].

Cats exposed to ectoparasites without preventive measures have higher exposure to VBP and will presumably experience higher rates of vector borne infections. Consequently, animal health experts routinely recommend ectoparasite control measures for dogs and cats. In the United States, the Companion Animal Parasite Council (CAPC) recommends flea and tick control administration for all 12 months of the year [4, 29]. Similarly, the European Scientific Counsel on Companion Animal Parasites (ESCCAP) advises that flea prevention can be necessary year-round with tick prophylaxis during the entire seasons in which ticks are active [5]. In a recent survey of veterinarians in the US, most recommended year-round use of a flea and tick medication for cats [30]. A survey of veterinarians in the US, UK and Australia recommended approximately 12 months of flea protection per year and 9–12 months of tick protection for dogs [31].

Ectoparasiticide products are labeled to treat just fleas or both fleas and ticks on cats. Most of these are dosed monthly by the pet owner and can be administered either orally or by topical application. One product containing the isoxazoline-class active ingredient fluralaner (BRAVECTO® TOPICAL SOLUTION, Merck Animal Health, Madison, NJ, USA), is labeled for re-administration to cats every 8–12 weeks therefore providing an extended period of protection compared to monthly re-treatments. Most ticks are treated with fluralaner with 12-week redosing, although the product is labelled for 8-week redosing when treating *Dermacentor variabilis*.

The effectiveness of any ectoparasite preventive medication is a function of the clinical efficacy of the medication and the actual use of the product by the end user. The end user is typically a cat owner, and owner behaviors that affect product efficacy include the timing of dose administration and accurate application when the dose is required.

Clinical efficacy is determined using well controlled experiments, with dose administration frequency used as recommended in precise accordance with label indications [32]. However, studies of dog owner adherence to veterinary recommendations indicate that actual field use of these products is typically not precise. Veterinarians tend to recommend year-round ectoparasite protection, however, product purchase records in studies evaluating owner purchase behavior found that ectoparasite medication purchases provided only 2.9–6.1 months of ectoparasite prevention when purchased by dog owners over a 12-month period [30, 31, 33, 34].

Some limited data are available regarding real-world ectoparasiticide purchases and use by cat owners. A survey of 312 dog and cat owners at the Small Animal Hospital of Veterinary Medicine, Lisbon University found that 63.6% of cats were treated with ectoparasiticides although most at infrequent intervals [35]. A retrospective study of 1,226 feline patients at a US veterinary teaching hospital found that only 38% of the cat population received ectoparasite preventatives with 18% receiving treatment year-round and 13% receiving treatment seasonally [36]. A study of cat owner adherence to veterinary recommendations on ectoparasite prevention using product purchase records found that over a 12-month period, cat owners purchased an average of 4.2 months of ectoparasiticide protection with fluralaner, 3.6 months with fipronil/(S)-methoprene/pyriproxyfen (FRONTLINE® Gold, Boehringer Ingelheim, Duluth, GA) or 2.8 months with imidacloprid/pyriproxyfen (ADVANTAGE® II, ELANCO, Greenfield, IN) [37]. These results indicate that the duration of pet owner adherence to veterinarian recommendations for ectoparasite control measure is far shorter than veterinarians recommend. The intrinsic efficacy of ectoparasiticide products can be excellent, however, the actual effectiveness observed by cat owners over the year will be much lower because there is treatment underutilization and repeated lapses in adherence to veterinarian recommendations.

This earlier research documents the number of doses purchased and the associated overall ectoparasite protection months provided during the year; however, these results do not tell the whole story of ectoparasite protection [33, 34, 37]. These studies do not provide results on the timing of administration of each dose during the year. Timing of administration affects the consistent protection that an ectoparasiticide product can deliver. To meet the veterinary recommendation, each dose is ideally given on time with no delay to deliver 12 contiguous months of ectoparasite protection. Certain ectoparasites, such as *Ctenocephalides felis felis* and *Ctenocephalides canis* live in the home environment year-round, and continuous, uninterrupted ectoparasite control measures are essential for successful elimination of established populations of these parasites. For example, successful flea control requires 2–3 months of continuous, timely administration of a highly efficacious flea product to drive an established flea infestation to extinction on the pet and within the home [38–43]. A missed or delayed ectoparasite protection dose creates a dosing gap that potentially allows the ectoparasite population to recover and re-establish. Therefore, gaps in therapy lead to treatment failures and frustration of well-intentioned pet owner efforts. Gaps in ectoparasite preventive product purchases provide evidence of administration delays and reduce owner-observed product effectiveness, potentially negatively impacting cat health.

The objective of this study was to evaluate gaps in owner administration of veterinary prescribed ectoparasiticide products in the United States by analyzing data on cat owner product purchases. This study also examines the impact of these purchase gaps on the overall period of ectoparasite protection and the proportion of time ectoparasite protection can be provided over a 12-month use period. Studies in human and veterinary medicine report significantly higher adherence rates for medications with a longer duration of action and decreased dosing frequency [44–49], therefore, this study also determines whether there is a difference in purchase gaps for owners prescribed a longer duration ectoparasiticide for their cat compared with owners prescribed shorter duration, monthly products.

## Methods

Cat owner product purchase records for ectoparasiticide medications sold at U.S. veterinary clinics were obtained and analyzed to determine purchase intervals and calculate gaps between dose purchases. Purchase gaps were used to estimate the extent of timely product administration. A purchase gap was declared if the subsequent product dose was purchased at a time after the period of efficacy of a previous dose had ended. Cat owners were assumed to have administered the product on the day of purchase, therefore, these data determine the minimum potential gap size. We were unable to assess a gap if the cat owner either purchased multiple doses at the same time or purchased one or more subsequent doses before completion of the period of efficacy of the previous dose. The data were also analyzed to calculate: the proportion of cat owners who purchased a single dose per year; the proportion who purchased multiple (> 1) doses per year without a detectable gap between doses; the proportion who purchased multiple doses with a gap between doses.

Retrospective topical feline ectoparasiticide purchase transaction data were obtained for individual cats from 671 veterinary clinics throughout the U.S. for the period January 1, 2017 through June 30, 2019. Data records were masked to conceal the identity of the veterinary clinic and cat owner by using unique numeric identifiers for clinic, client and pet that allowed purchase records to be associated for individual owners and cats. Some cat demographic data, including age and body weight, were collected. Each ectoparasiticide medication purchase data entry included a date, a product description—including packaging and doses—and the quantity of product purchased on that date. Purchases could include single packs or multi-packs for each product. Transaction records included were for ectoparasite medication sales made by the clinic to the client in the name of a single patient. Ectoparasiticide products included were limited to those that are topically applied and frequently prescribed within the database. Included were fluralaner topical solution, imidacloprid (with and without pyriproxyfen), fipronil/S-methoprene/pyriproxyfen and selamectin (REVOLUTION®, Zoetis, Kalamzoo, MI).

Only transactions by clients who were “pure users”, meaning that they did not have purchases from multiple brands, were included in the study. The study period for each client was defined as the 12 months following their initial product purchase date. Only cats with purchase records available for the 12 months following the initial purchase, regardless of whether more product was purchased or not, were included. Purchases were excluded from the analysis if the transaction record indicated purchase of more than 24 months of doses in a 12-month period because these indicate bulk purchase or purchase of products for more than one cat. Product returned for credit and purchases for animals other than cats were also excluded. Fluralaner topical solution has a 12-week dosing interval for most label indicated ectoparasites, therefore this product could have a maximum of 5 purchases within a 12-month period, while the monthly products could have a maximum of 12 purchases within a 12-month period. Fluralaner dosed per label directions to protect against *D. variabilis* has an 8-week dosing interval and would be dosed with a maximum of 7 purchases within a 12-month period.

Gaps were calculated between doses rather than between purchases of the product, because a purchase could lead to acquisition of one dose or multiple doses. An algorithm was used to process each purchase record in the original dataset so that a novel dataset was produced as follows: if a purchase record was for a single dose, the purchase date became the “administration” date for the dose and the protection end date was calculated based on the product duration; if a purchase record was for multiple doses, the administration date of the first dose was the purchase date and the protection end date was calculated from the number of doses multiplied by the product protection period for each dose. If there were multiple purchase dates, with different product dose quantities on each purchase date, then the algorithm was applied similarly. The analysis assumes that single doses were administered to the cat when purchased or at the correct consecutive interval when multiple doses were purchased in a single transaction. This assumption leads to a best-case estimate of the gap between doses. Owners may, in reality, have waited or deferred giving either the first or subsequent dose, creating a larger gap than detected in this analysis.

The total number of doses was calculated for each cat for the entire 12-month period. In addition, for each dose purchased during the 12-month period there is an associated administration date, and a protection end date calculated using the re-administration interval per the product label for the purchased product. This subsequently enabled calculation of the gap (in days) between the end date of the ectoparasite protection provided by the first administered dose and the recorded administration date for the next dose. A gap was not detectable when the cat owner purchased multiple doses at the same time. Based on this method a matrix was developed for all possible dose gap possibilities. For example, for fluralaner, the possible gaps during the 12 months could be “Dose 1–2”, “Dose 2–3”, “Dose 3–4” and “Dose 4–5” while for monthly products, there could be possible dose gaps starting with “Dose 1–2”, “Dose 2–3” continuing up to “Dose 11–12”.

**Fig. 1:**
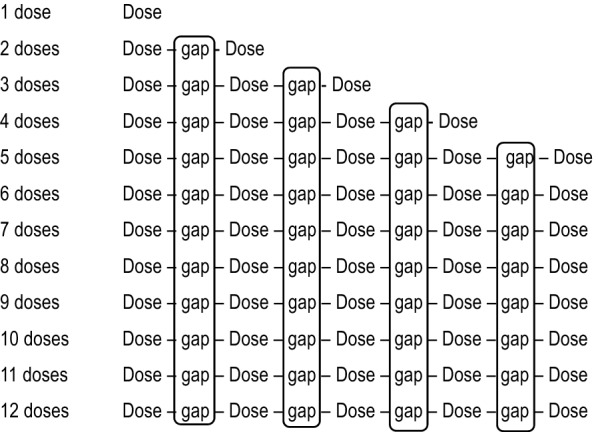
Method for calculating average gaps between ectoparasiticide purchases of 1–12^+^ doses.^* *^Average gaps were calculated using purchase data that demonstrated purchase gaps. ^+^ Because fluralaner topical has a 12-week dosing interval for most parasites on the product label, a maximum of 5 doses fluralaner topical purchases were considered for the 12-month period studied and 12 doses of the monthly products

The dose gaps described above, e.g. “Dose 1–2”, were combined across all of the annual purchased doses for each product, then used to calculate the average gap (weeks) (Fig. 1). Each possible gap was calculated separately and enabled calculation of gap totals for each different dose, gap totals by total doses, and average gaps for each product.

The “doses plus gap period” is defined as the time duration encompassing the dosing intervals for two consecutive doses plus the average gap between purchases of these doses. The period of ectoparasite protection could then be calculated as the percent of the time during the doses plus gap period when ectoparasite protection is available.

Statistical summaries (mean, median, standard error, range) were calculated for age and weight of cats prescribed each study product.

## Results

Ectoparasiticide purchase records were obtained for 114,853 cats from 671 veterinary clinics. Regional distribution of participating clinics was: 50% Southeast (*n* = 341), 21% Mid-west (*n* = 142), 15% Southcentral (*n* = 98), 8% West (*n* = 51) and 6% Northeast (*n* = 39). Average cat age was 6.4 years (range 0.5–25.0 years) and the average weight was 4.3 kg (range 0.1–13.6 kgs). Product details for the four feline ectoparasiticide products examined are included in Table 1.

**Table 1:** Information on topical flea and tick control products used in a study of administration gaps

Brand name	Manufacturer	Active ingredients	Indications			Redosing interval
			Fleas	Ticks	Heartworm, hookworm, roundworm	
BRAVECTO Topical Solution for Cats	Merck Animal Health	Fluralaner	X	X		12 weeks (or 8 weeks if *D. variabilis* is of concern)
FRONTLINE Gold for Cats	Boehringer Ingelheim	Fipronil/(S)-methoprene/pyriproxyfen	X	X		Monthly
ADVANTAGE/ Advantage II	ELANCO Animal Health	Imidacloprid/Imidacloprid/ pyriproxyfen	X			Monthly
REVOLUTION	Zoetis	Selamectin	X		X	Monthly

Most cat owners purchased less than 6 months of ectoparasite protection regardless of the product used, with the majority purchasing just 1–3 months of protection while a smaller proportion of cat owners purchased 7–12 months of protection (Tables 2 and 3).

**Table 2:** Proportional purchases of select ectoparasiticides

Owner Yearly Purchases	Fluralaner (*n* = 27,138)	Fipronil/(S)-methoprene/pyriproxyfen (*n* = 10,171)	Imidacloprid/pyriproxyfen (*n* = 3480)	Selamectin (*n* = 74,064)
1–6 months/year	87%	86%	91%	77%
1–3 months/year	67%	67%	75%	56%
4–6 months/year	20%	19%	16%	21%
7–12 months/year	13%	14%	9%	23%

**Table 3:** Ectoparasiticide doses purchased by cat owners during a 12-month period

Doses purchased per 12 months	Fluralaner^a^ (*n* = 27,138)	Fipronil /(s)-methoprene/ pyriproxyfen (*n* = 10,171)	Imidacloprid/pyriproxyfen (*n* = 3480)	Selamectin (*n* = 74,064)
1	18,265 (67.3%)	3609 (35.5%)	1903 (54.7%)	28,044 (37.9%)
2	5216 (19.2%)	1342 (13.2%)	467 (13.4%)	9202 (12.4%)
3	1986 (7.3%)	1846 (18.2%)	239 (6.9%)	8355 (11.3%)
4	1264 (4.7%)	963 (9.5%)	271 (7.8%)	2882 (3.9%)
5	407 (1.5%)	211 (2.1%)	67 (1.9%)	1392 (1.9%)
6		1045 (10.3%)	297 (8.5%)	12,160 (16.4%)
7		141 (1.4%)	37 (1.1%)	1584 (2.1%)
8		411 (4.0%)	94 (2.7%)	3118 (4.2%)
9		264 (2.6%)	16 (0.5%)	1595 (2.2%)
10		50 (0.5%)	12 (0.3%)	526 (0.7%)
11		38 (0.4%)	8 (0.2%)	361 (0.5%)
12		251 (2.5%)		4845 (6.5%)

^a^Because fluralaner topical has a 12-week dosing interval for most parasites on the product label, a maximum of 5 doses fluralaner topical purchases were considered for the 12-month period studied (Note: Up to 7 doses of fluralaner may be required for full year protection in areas where *Dermacentor variabilis* are of concern)

Approximately 45% (*n* = 51,821) of cat owners purchased just one ectoparasiticide dose in a 12-month period. Of owners who purchased fluralaner, 67.3% (*n* = 18,265) bought one dose which provides each cat with 12 consecutive weeks of ectoparasiticide protection in a year. The proportion of owners who purchased just one dose of a monthly product were 35.5% (*n* = 3609) for fipronil/(S)-methoprene/pyriproxyfen, 54.7% (*n* = 1903) for imidacloprid/pyriproxyfen and 37.9% (*n* = 28,044) for selamectin, respectively. These cats would receive just 4.3 consecutive weeks of protection in a 12-month period (Table 3).

The percent of purchases made with or without a gap were determined for cat owners who purchased more than 1 dose in the 12-month period, and then average gap length was calculated for each inter-dose interval including doses 1–2, 2–3 … and so on (see Fig. 1) (Table 4).

**Table 4:** Cat owner ectoparasiticide purchases with and without gaps

Doses purchased	Fluralaner^a^ (*n* = 27,138)	Fipronil/s)-methoprene/pyriproxyfen (*n* = 10,171)	Imidacloprid/pyriproxyfen (*n* = 3480)	Selamectin (*n* = 74,064)
1 dose Total *N* (% of total)	18,265 (67%)	3609 (35%)	1903 (55%)	28,044 (38%)
2–12 doses (%)	(33%)	(65%)	(45%)	(62%)
Total purchasing > 1 dose	8873	6562	1577	46,020
No Gap N (%)	3699 (42%)	4950 (75%)	975 (62%)	31,587 (69%)
urchase Gap N (%)	5174 (58%)	1612 (25%)	602 (38%)	14,433 (31%)
Purchased ≥ 2 doses	8873	6562	1577	46,020
Total with 1–2 dose gap	4301	848	395	8429
% with gap	48%	13%	25%	18%
Ave. Gap (Weeks)	11.2	13.9	13.3	12.9
Purchased ≥ 3 doses	3657	5220	1110	36,818
Total with 2–3 dose gap	1730	485	238	4183
% with gap	47%	9%	21%	11%
Ave. Gap (Weeks)	7.7	12.2	10.0	10.2
Purchased ≥ 4 doses	1671	3374	871	28,463
Total with 3–4 dose gap	647	373	113	3262
% with gap	39%	11%	13%	11%
Ave. Gap (Weeks)	4.5	12.7	10.2	10.2
Purchased ≥ 5 doses	407	2411	600	25,581
Total with 4–5 dose gap	103	239	107	1625
% with gap	25%	10%	18%	6%
Ave. Gap (Weeks)	2.5	10.1	11.1	8.2
Purchased ≥ 6 doses		2200	533	24,189
Total with 5–6 dose gap		79	37	833
% with gap		4%	7%	3%
Ave. Gap (Weeks)		6.6	5.7	5.6
Purchased ≥ 7 doses		1155	236	12,029
Total with 6–7 dose gap		134	36	2164
% with gap		12%	15%	18%
Ave. Gap (Weeks)		10.0	9.0	9.1
Purchased ≥ 8 doses		1014	199	10,445
Total with 7–8 dose gap		42	7	428
% with gap		4%	4%	4%
Ave. Gap (Weeks)		5.9	3.0	4.3
Purchased ≥ 9 doses		603	105	7327
Total with 8–9 dose gap		35	8	361
% with gap		6%	8%	5%
Ave. Gap (Weeks)		6.2	3.3	5.4
Purchased ≥ 10 doses		339	89	5732
Total with 9–10 dose gap		23	5	276
% with gap		7%	6%	5%
Ave. Gap (Weeks)		5.6	5.6	4.4
Purchased ≥ 11 doses		289	77	5206
Total with 10–11 dose gap		5	3	131
% with gap		2%	4%	3%
Ave. Gap (Weeks)		3.8	2.3	3.2
Purchased 12 doses		251	69	4845
Total with 11–12 dose gap		2	1	40
% with gap		1%	1%	1%
Ave. Gap (Weeks)		2.0	1.0	2.7

^a^Because fluralaner topical has a 12- week dosing interval for most parasites on the product label, a maximum of 5 doses fluralaner topical purchases were considered for the 12-month period studied

**Fig. 2:**
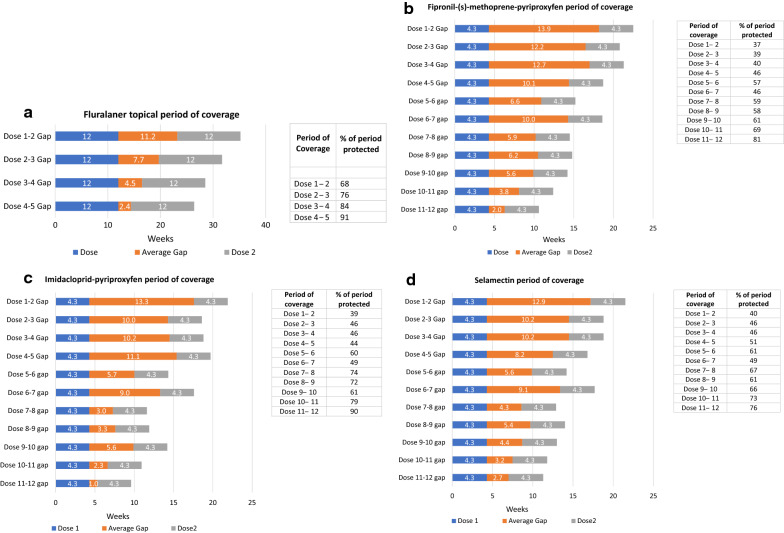
Duration of each 2-dose period of coverage, including gap, and % ectoparasite protection

The proportion of cat owners who purchased more than 1 dose of their respective product with at least 1 gap of any duration between doses was 58% (fluralaner), 25% (fipronil/(s)-methoprene/pyriproxyfen), 38% (imidacloprid/pyriproxyfen), and 31% (selamectin) (Table 4). The fraction of purchases with a gap between doses and the average length of the purchase gap generally decreased as cat owners purchased more doses per year. Cat owners who purchased fluralaner often had the shortest purchase gaps between each of the doses given in the 12-month period (Fig. 2a–c).

Ectoparasiticide purchase gaps lead to periods of time when cats do not have ectoparasite protection. This creates a time of increased risk for infestation and exposure to VBP. Therefore, for each product, the duration of each “doses plus gap period” was determined (Fig. 2a–c) and the percentage of time during that period when ectoparasiticide protection could be available, assuming timely administration, was calculated (Table 5 and Fig. 2a–c). For cat owners prescribed fluralaner, the percentage of time when ectoparasiticide was available within each “2 dose plus gap period” gradually increased and was 68% for 1–2 doses, 76% for 2–3 doses, 84% for 3–4 doses and 91% for 4–5 doses (Table 5 and Fig. 2a). The percentage of time when ectoparasiticides were available within each “doses plus gap period” for the monthly products was less than for fluralaner at all comparable dose intervals. For fipronil/(s)-methoprene/pyriproxyfen users, the percentage of ectoparasite protection for each dosing period was 38%, 41%, 40%, 46% and 57% for dose periods of use 1–2, 2–3, 3–4, 4–5, and 5–6, respectively and rose to 81% by the 12^th^ dose (Table 5 and Fig. 2b). For imidacloprid/pyriproxyfen, the percentage of ectoparasite protection for each dosing period was 39%, 46%, 46%, 44%, and 60% for doses 2–6 respectively and rose to 90% by the 12^th^ dose (Table 5 and Fig. 2c). For cats that received selamectin, the percentage of ectoparasite protection for each dose plus gap period was 40%, 46%, 46%, 51% and 61%, for dose periods of use 1–2, 2–3, 3–4, 4–5, and 5–6, respectively, and rose to 76% by the 12^th^ dose (Table 5 and Fig. 2d).

**Table 5:** The proportion of each 2-dose interval with and without ectoparasite protection

Product	Fluralaner topical			Fipronil/(s)-methoprene/pyriproxyfen			Imidacloprid/pyriproxyfen			Selamectin		
	Doses plus gap period^a^ (weeks)	% of time protected	% of time not protected	Period of coverage^a^ (weeks)	% of time protected	% of time not protected	Period of coverage^a^ (weeks)	% of time protected	% of time not protected	Period of coverage^a^ (weeks)	% of time protected	% of time not protected
Dose period												
Dose 1–2	35.2	68	32	22.5	38	62	21.9	39	61	21.5	40	60
Dose 2–3	31.7	76	24	20.8	41	59	18.6	46	54	18.8	46	54
Dose 3–4	28.5	84	16	21.3	40	60	18.8	46	54	18.8	46	54
Dose 4–5	26.5	91	9	18.7	46	54	19.7	44	56	16.8	51	49
Dose 5–6				15.2	57	43	14.3	60	40	14.2	61	39
Dose 6–7				18.6	46	54	17.6	49	51	17.7	49	51
Dose 7–8				14.5	59	41	11.6	74	26	12.9	67	33
Dose 8–9				14.8	58	42	11.9	72	28	14.0	61	39
Dose 9–10				14.2	61	39	14.2	61	39	13.0	66	34
Dose 10–11				12.4	69	31	10.9	79	21	11.8	73	27
Dose 11–12				10.6	81	19	9.6	90	10	11.3	76	24

^a^“Doses plus gap period” is the time from the administration of one dose through the end of the treatment period of the subsequent dose, including the dosing gap, measured in weeks. For Fipronil/(s)-methoprene/pyriproxyfen , imidacloprid/pyriproxyfen and selamectin, the single dose duration of efficacy was 4.3 weeks. For fluralaner topical the single dose duration of efficacy was 12 weeks per label indication (Note: Up to 7 doses of fluralaner may be required for full year protection in areas where *Dermacentor variabilis* are of concern)

**Fig. 3:**
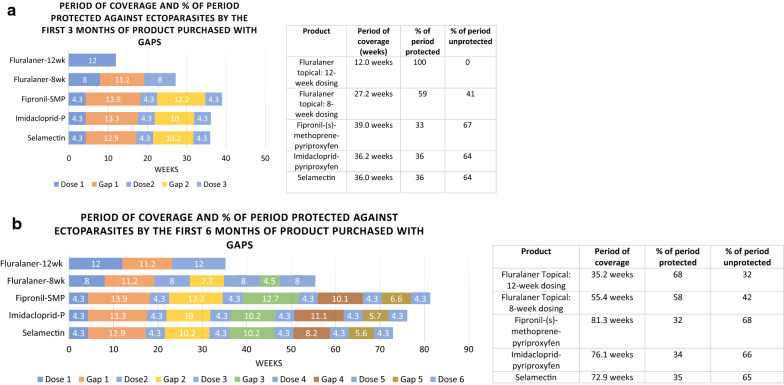
Impact of purchase gap on the period of protection

The impact of purchase gaps on the percentage of time that ectoparasite protection was available is shown for owners who purchased 1–3 months and 1–6 months of medication in a year, the most common protection durations purchased (Fig. 3a, b). The fluralaner dosing interval for most ectoparasites indicated is 12 weeks, therefore 1 and 2 doses of fluralaner were compared to 3 and 6 doses of the monthly duration products fipronil/(s)-methoprene/pyriproxyfen, imidacloprid/pyriproxyfen and selamectin. Because fluralaner is also labeled for a 8-week dosing interval when necessary for the control of *D. variabilis,* 2 and 4 doses of fluralaner were also compared to 3 and 6 doses of the monthly duration products. The total duration of the 3-month and 6-month “doses plus gap periods” for each product are shown (Fig. 3a, b) and for each of these periods the percentage of time when ectoparasiticide protection could be available was determined and compared.

These comparisons show that fluralaner, with its extended duration dosing interval and shorter purchase gaps, consistently provided longer ectoparasite protection for a larger proportion of each of the “doses plus gap” periods (Fig. 3a, b). The percentage of ectoparasite protection available during use of the first 12 weeks or 3 months of product purchased was 100% for fluralaner with the 12-week dosing interval (12 weeks or 2.8 months) and 59% with the 8-week dosing interval, 33% for fipronil/(s)-methoprene/pyriproxyfen, 36% for imidacloprid/pyriproxyfen and 36% for selamectin. The percentage of time that ectoparasite protection was available when cat owners purchase 6 months of protection, two doses of fluralaner administered at 12 week intervals or 4 doses fluralaner administered at 8 week intervals or 6 months of the monthly products was 68% for fluralaner administered with 12-week intervals, 58% for fluralaner with the 8-week dosing interval, 32% for fipronil/(s)-methoprene/pyriproxyfen, 34% for imidacloprid/pyriproxyfen and 35% for selamectin.

## Discussion

Timely administration of ectoparasiticides consistent with veterinarian recommendations and product labeling is essential for optimal efficacy and effective ectoparasite control. Prior studies have made clear that pet owners fall short of meeting their veterinarians’ recommendations for ectoparasite protection by purchasing fewer months per year of ectoparasite protection than advised [30, 31, 33, 34, 37]. The current study expands our understanding of this adherence problem by not only looking at how much flea and tick medication they purchase in a year, but how they purchase it. Examining gaps in ectoparasite product purchases can be used to estimate the extent of delayed administration and its impact on the proportion of time ectoparasite protection might be provided. The method of looking at gaps in purchases identifies the minimum amount of time that can occur between the administration of consecutive doses. Delay in administration or forgetting to administer the medication could create gaps that are substantially greater than our model proposes.

Cat owners who have gaps between purchases were able to provide more weeks of consecutive medication by using an extended duration product. Not only was the period of efficacy long enough to resolve a flea infestation, fluralaner provided more consistent medication delivery through shorter average gaps between doses.

Cat owners purchase less ectoparasite prevention medication each year than is recommended by veterinarians [30, 31, 33, 34, 37]. In this study, most cat owners purchased 1–3 months of protection per year. When we look at ectoparasite medication purchases with gaps between doses in owners who bought 2 or more doses, gaps were seen in 25–58% of purchases. Within a brand, the average gap got smaller as cat owners bought more doses per year. Gaps between doses impact the effectiveness of flea medications because successful flea control requires 2–3 months of continuous, on-time administration of an efficacious ectoparasiticide in order to eliminate an established flea infestation on the pet and within the home [38–43]. Measurement of ectoparasite product purchase gaps provides an estimate of the extent of administration delays, along with assessment of the proportion of time when cats have or don’t have protection against ectoparasites.

The gaps between purchases of ectoparasiticide doses seen in this study are a statement of the minimum possible amount of time between consecutive dose administrations. The reality for some owners is that the doses are given with some delay after dose purchases, meaning that the gap in time for administration of follow-up doses are actually larger than were seen here.

Human patients and pet owners often acquire all of the prescribed medication for a period of time in one purchase. In this study, over half (57%) of cat owners purchased multiple doses of ectoparasiticide at one time, and thus may have had purchase gaps that could not be identified. The administration of medication is not observed once it goes home and some proportion is certainly associated with delayed and missed administration. Studies in both human and veterinary medicine have shown that timely administration may not occur even when medication is dispensed in full [35, 44, 45, 47, 48, 50] and up to 50% of chronic disease medications given to human patients were not taken as prescribed [51].

For ectoparasite product purchases with a measurable gap, this gap represents an interruption in redosing and leads to a lack of ectoparasite protection and possibly, perceived product failure.

When pet owners purchased ectoparasiticide medication with a gap, this gap was consistently smaller and the protection time percentage was larger for fluralaner than for the monthly products. Fluralaner purchase led to cats getting a proportional protection in their “doses plus gap period” that ranged up to 91% for any 2-dose use periods in 12 months. One fluralaner dose is sufficient protection duration to eliminate a flea infestation in a household. Monthly products require three consecutive doses with no inter-dose gap to be similarly effective. Purchase profiles and more frequent redosing intervals of monthly administered products led to a time of ectoparasite protection that was often less than half that of fluralaner.

Comparisons of these ectoparasiticides and their purchase gaps across the most commonly purchased number of doses, (3-months and 6-months) show that the proportion of the corresponding period that included ectoparasite protection was consistently greater for fluralaner than for the monthly products, when dosed at either the 12-week or 8-week redosing intervals. This is in part because extended duration fluralaner does not need to be redosed as often as the monthly medications, and failure to redose reduces the total amount of time when ectoparasite protection is available. Extended duration medication also allows fewer opportunities for purchase gaps, a reduced chance of missing a dose and thereby a greater period of ectoparasite protection. Considering owners who purchased 6 months of ectoparasiticide with gaps, cats receiving fluralaner had a proportional protection period nearly twice as long during the 6 month period compared to cats receiving monthly ectoparasiticides; 68% for fluralaner with 12-week redosing versus 32%, 34% and 35% for fipronil/(s)-methoprene/pyriproxyfen, imidacloprid/pyriproxyfen, and selamectin, respectively (Fig. 3b).

Inconsistent ectoparasiticide administration (failure to administer the product at correct intervals or to administer the product at all) in the face of continued parasite exposure is one explanation for parasite lack of efficacy (LOE) reports [52]. Any gap in the delivery of a monthly duration product is unacceptable when there is a need to deliver the 2–3 months of continuous treatment to eliminate a household flea infestation. One benefit of extended duration flea and tick medication is the duration of effectiveness without redosing [43, 53].

Dosing gaps not only impact the effectiveness of ectoparasiticide medications but they can also impact the number of months of coverage that owners purchase in the following year. Cat owners who purchase multiple doses of ectoparasiticides and also have sizeable gaps between doses may not be able to use all of their medication within 12 months. The cumulative effect of multiple dosing gaps may be one reason that owners would report in a wellness visit that they don’t need more flea and tick medication in the new year because they have product left over from the previous year.

Getting pet owners to adhere to veterinary ectoparasite protection recommendations is not easy. Studies on human patient adherence to prescribed treatment regimens have shown that simpler, less frequent dosing regimens improve patient compliance across a variety of therapeutic classes [44–46, 49, 50, 54–57]. In veterinary medicine, adherence to veterinary prescribed dosing intervals tends to be better when dosing is less frequent with extended dosing intervals [47, 48]. Studies of dog and cat owner flea and tick control adherence over a 12-month period show that owners who purchase a longer duration flea and tick product protected their pets for more months of the year than owners who purchased a monthly treatment [33, 34, 37]. The present study similarly demonstrates a benefit of the longer duration flea and tick product, fluralaner. Cat owners who had gaps between dose purchases but chose the longer duration product provided more weeks of consecutive medication necessary for ectoparasite infestation resolution, had shorter gaps between doses, and an increase in the overall percentage of time with effective ectoparasite protection during each 2-dose use period than cat owners who purchased a monthly treatment.

Use of a longer duration medication reduces treatment interruptions which should improve the overall effectiveness and result in decreased exposure to VBP while providing greater satisfaction with efforts to remove ectoparasites.

In spite of the size of the population examined, this study was limited by the relatively small number of cat owners who purchased more than 6 months of ectoparasite control The percent period of protection was assessed for fluralaner at 8-week and 12-week redosing intervals showing up to 6 months of protection, which is the most commonly purchased number of multiple dose purchases for all products examined. In addition, on-time administration of ectoparasiticides purchased in multi-dose transactions could not be confirmed. Finally, some cat owners may purchase some products through non-veterinary channels (over-the-counter sales or online pharmacies) that are not captured in this transaction database analysis and thus could supplement the doses purchased from veterinarians.

## Conclusions

Cat owners fail to adhere to veterinary ectoparasiticide protection recommendations because they often purchase only 1–3 months of protection per year (56–75%) and frequently have gaps between ectoparasiticide dose purchases (25–58% who purchase 2 doses or more per year). Gaps between doses of ectoparasiticidal products could lead to treatment failures and owner frustration with ectoparasite control. On average, an extended duration flea and tick treatment achieves more consecutive months of ectoparasite protection. Each 12-week dose of fluralaner delivers a longer (up to 3X) period of medication activity without re-dosing, compared to a monthly ectoparasiticide product. While the current ectoparasiticide products have proven efficacy against fleas ± ticks, their effectiveness is greatly impacted by dosing gaps. The use of an extended duration ectoparasiticide and the reduced need for re-dosing reduces the impact of missed or delayed doses on annual ectoparasite protection.

## Data Availability

The datasets were obtained from a public source (VetInformatics, Inc., Rolling Meadows, Ill, USA). The analysis was generated during the current study and is not publicly available because this is the proprietary property of Merck & Co., Inc., Kenilworth, NJ, USA.

## Abbreviations

CAPC: Companion Animal Parasite Council
ESCCAP: European Scientific Counsel on Companion Animal Parasites
OTC: Over-the counter
VBP: Vector borne pathogens
